# Comparison between recommended and mandatory vaccine uptake during adolescence in Italy

**DOI:** 10.1093/eurpub/ckac130.006

**Published:** 2022-10-25

**Authors:** MR Caracciolo, S Angelillo, F Ficara, D Venturi, F Licata, A Bianco

**Affiliations:** Department of Health Sciences, University of Catanzaro “Magna Græcia”, Catanzaro, Italy

## Abstract

**Background:**

Immunization programs are key preventive interventions and have largely contributed to reducing the burden of infectious diseases and decreasing related morbidity, mortality and healthcare costs. The study aimed to investigate coverage regarding diphtheria, tetanus, and acellular pertussis (dTap) - Inactivated Poliomyelitis Vaccine (IPV) and Human Papilloma Virus (HPV) vaccine and attitudes towards vaccinations among undergraduate university students in Southern Italy.

**Methods:**

This cross-sectional study was conducted among 327 students through an anonymous online questionnaire and included socio-demographic characteristics, attitudes towards vaccinations overall and specifically on dTap-IPV and HPV, reasons for having received or not vaccinations and willingness to receive vaccinations.

**Results:**

One third of the students were concerned about serious adverse effects of vaccines and 95% believed that vaccines for uncommon diseases are useless. During adolescence, 89% of the sample received the mandatory dTap-IPV vaccine booster. Among unvaccinated students, 45% were unwilling to get vaccinated against dTap-IPV because they believed not to be at risk of infection (59%) and had lack of recommendation (35.3%). Regarding vaccination against HPV, 67% had received the recommended schedule. Among those who did not receive it, 34% were unwilling to get vaccinated because they did not feel at risk of HPV infection (41%). Interestingly, 16% of the sample disclosed some barriers to access vaccination centers. Moreover, 30% declared that HPV vaccination was discouraged by healthcare professionals (HCPs).

**Conclusions:**

Vaccination uptake is worryingly low and national objective coverage seems not still achieved. Likewise, risk perception of vaccine-preventable diseases was low and it seems negatively impact on the intention to get vaccinated. Improving vaccine confidence among HCPs is crucial as they have been shown to have the potential to influence patient vaccination uptake.

**Key messages:**

- Skilled communication with a trusted HCP could increase acceptance of vaccine during adolescence and address vaccine hesitancy.
- Strategies to disseminate information on vaccines should be established to increase mandatory and recommended vaccines coverage.

